# Supplementary material for: Asynchronous magnetic resonance elastography: Shear wave speed reconstruction using noise correlation of incoherent waves
Source: Magn Reson Med. 2022 Oct 27;89(3):990–1001. doi: 10.1002/mrm.29502 (PMC9792433; doi:10.1002/mrm.29502)
Supplement: Supplementary file 1 — DATA S1 MATLAB code used to generate the numerical simulations of Figure 1 [file MRM-89-990-s002.zip › k-Wave/helpfiles/k-wave_function_ref_alph.html]

Functions - Alphabetical List (k-Wave)


# Functions — Alphabetical List

 View Functions By Category

A  
B  
C  
D  
E  
F  
G  
H  
I  
J  
K  
L  
M  
N  
O  
P  
Q  
R  
S  
T  
U  
V  
W  
X  
Y  
Z  
  

acousticFieldPropagator  
acousticFieldPropagatorC  
addNoise  
applyFilter  
angularSpectrum  
angularSpectrumCW  
attenComp  
beamPlot  
benchmark  
bioheatExact  
calculateMassSource  
calculateMassSourceCW  
cart2grid  
checkFactors  
checkStability  
createCWSignals  
db2neper  
envelopeDetection  
expandMatrix  
extractAmpPhase  
filterTimeSeries  
findClosest  
fitPowerLawParams  
flyThrough  
focus  
focusedBowlONeil  
fourierShift  
fwhm  
gaussian  
gaussianFilter  
getAlphaFilter  
getBLI  
getColorMap  
getComputerInfo  
getDateString  
getFDMatrix  
getkWavePath  
getOptimalPMLSize  
getSpacedPoints  
getWin  
gradientFD  
gradientSpect  
grid2cart  
h5compare  
hounsfield2density  
interpCartData  
interpftn  
kspaceFirstOrder1D  
kspaceFirstOrder2D  
kspaceFirstOrder2DC  
kspaceFirstOrder2DG  
kspaceFirstOrder3D  
kspaceFirstOrder3DC  
kspaceFirstOrder3DG  
kspaceFirstOrderAS  
kspaceFirstOrderASC  
kspaceLineRecon  
kspacePlaneRecon  
kspaceSecondOrder  
kWaveDiffusion  
kWaveGrid  
kWaveTransducer  
loadImage  
logCompression  
makeArc  
makeBall  
makeBowl  
makeCartCircle  
makeCartSphere  
makeCircle  
makeDisc  
makeLine  
makeMultiArc  
makeMultiBowl  
makeSphere  
makeSphericalSection  
maxND
